# 

**Published:** 1881-03-20

**Authors:** 


### TABLE OF CONTENTS.

ORIGINAL AND SELECTED ARTICLES.

Conversations upon the Physical and Mental Hygiene of Girlhood, by T. S. Powell, M.D, Atlanta, 81. Operation for Strictured Hernia, by T. H. Caldwell, M.D., 91. Alcoholic Mania in Connection with Acute Disease, 92. Atomization in Pulmonary Hemorrhage, 96.

ABSTRACTS AND GLEANINGS.

The Opium Habit 98. Minor Anæsthetics, 100. Recent Studies on the Nature of Malaria, 101. An Interesting Case from Practice, 103. The Use of Anæsthetics in Labor, 104. Syphilitic Ulceration of Velum accompanied with a Large Perforation. 106. Iodine as a Specific in Croupous Pneumonia, 107. The Prevalence of Leprosy in the United States; Report on Yellow Fever in the U. S. S. Plymouth, 108. Dermic Dressing in Variola; Fuchsine in Bright's Disease; Treatment of Asthma with the Induced Current, 109. On Cholera Infantum, Pilocarpin in Diphtheria, 110.

SCIENTIFIC ITEMS.

Luminous Paint; A "National" Radiant, 111. Toughened Lamp Chimneys; Preserving Wood; Providence of Bees; A New Oxide of Boron, 112.

PRACTICAL NOTES AND FORMULÆ.

For Vomiting in Pregnancy; Jamaica Dogwood in Treatment of Pertussis; Dysmenorrhœa; Removal of Nævus. 113. Chemistry of Common Life; Gastritis; Acute Catarrh; A Stimulating Expectorant; Special Affection Following Diphtheria, 114.

EDITORIAL AND MISCELLANEOUS.

Southern Medical College Commencement, 115. Catalogue of Members American Med. Association, 117. New York Academy of Medicine; American Medical Association, 119. Pamphlets Received; Receipts; Special Notices, 120.

